# Effects of Different Rearing Systems on Lueyang Black-Bone Chickens: Meat Quality, Amino Acid Composition, and Breast Muscle Transcriptome

**DOI:** 10.3390/genes13101898

**Published:** 2022-10-19

**Authors:** Shuya Zhang, Jiqiao Zhang, Chang Cao, Yingjie Cai, Yuxiao Li, Yiping Song, Xiuyu Bao, Jianqin Zhang

**Affiliations:** College of Animal Science and Technology, Northwest A&F University, Xianyang 712100, China

**Keywords:** Lueyang black-bone chicken, rearing system, meat quality, transcriptome, different expressed genes

## Abstract

The quality of poultry products depends on genotype, rearing system, and environment. The aim of this study was to investigate the effects of different rearing systems on meat quality, amino acid composition, and breast muscle transcriptome from Lueyang black-bone chickens. Lueyang black-bone chickens (*n* = 900) were randomly divided into three groups (cage, flat-net, and free-range groups), with three replicates per group (100 chickens per replicate). At 16 weeks, a total of 36 healthy chickens (six males and six females per group) were collected, and their breast muscles were sampled to detect meat quality parameters, amino acid composition, and fatty acid contents. Furthermore, breast muscles from six random hens in each group were used for RNA-seq analysis. The results revealed that the values of pH, shear force, inosine monophosphate (IMP), palmitic acid, and linoleic acid in the free-range group were significantly higher than those in the caged group (*p* < 0.05). Fat content in the free-range group was significantly lower than in the caged and flat-net groups (*p* < 0.05). Glutamate (Glu) levels, the amino acid crucial for the umami taste, was significantly higher in the free-range group than in the caged group (*p* < 0.05). Meanwhile, there was no significant difference between the free-range and flat-net groups (*p* > 0.05). The breast muscle transcriptome results showed that there were 291, 131, and 387 differently expressed genes (DEGs) among the three comparison groups (caged vs. free-range, flat-net vs. caged, and flat-net vs. free-range, respectively) that were mainly related to muscle development and amino acid metabolism pathways. To validate the accuracy of the transcriptome data, eight genes *(GOS2*, *ASNS*, *NMRK2*, *GADL1*, *SMTNL2*, *SLC7A5*, *AMPD1*, and *GLUL*) which relate to fat deposition, skeletal muscle function, and flavor formation were selected for Real-time Quantitative PCR (RT-qPCR) verification. In conclusion, these results suggested that rearing systems significantly influenced the meat quality and gene expression of Lueyang black-bone chickens. All the data proved that free-range and flat-net systems may provide better flavor to consumers by affecting the deposition of flavor substances and the expression of related genes. These findings will provide a valuable theoretical basis for the rearing system selection in the poultry industry.

## 1. Introduction

Lueyang black-bone chickens, a slow-growing broiler breed, are mainly distributed in Lueyang, Shaanxi, China [1]. During the long history of breeding and domestication, it has been raised around the hilly areas of Lueyang County, which helped it to form excellent meat quality and unique genetic characteristics [2]. It is the largest indigenous black-bone chicken breed in China and has Silkie-like dermal hyper-pigmentation, where the whole body is black, including the feathers, wingtips, beak, comb, skin, bones, legs, and paws [3]. In oriental countries, black-bone chickens are considered to contain nutritional and medicinal properties, and are an important component of medicinal diets [4].

Different rearing systems used for animals can affect welfare, growth performance, meat quality, stress resistance, and product price [5]. Currently, chicken-rearing systems in the Lueyang area mainly include cage rearing, flat-net rearing, and free-range rearing. The intensive, high-density caged system can effectively increase production efficiency. In contrast, the free-range system can improve welfare and enhance the product quality of poultry by increasing the amount of exercise, reducing the stocking density, and expanding the activity space [6]. For slow-growing broilers, previous studies have indicated that the free-range group performed better in terms of nutritional, food safety, and sensory properties, while the caged group had better growth performance, such as body weight and slaughter rate [7]. In general, slow-growing free-range chickens are more likely to meet consumer expectations for organic products because they exhibit less fat, have better flavors, and contain more polyunsaturated fatty acids (PUFAs) [8]. There are many studies on rearing systems on the meat quality of other breeds, but few studies on the excellent local breed of Lueyang black-bone chickens. Therefore, the main purpose of this study was to investigate the effects of different rearing systems on the meat quality of Lueyang black-bone chickens.

Meat quality is a complex trait that can be regulated by rearing factors, such as final slaughter weight and age, rearing environment, climatic conditions, and physical exercise [9]. Bogosav et al. [10] reported that free-range poultry products are superior to cage-reared products in tenderness and meat quality, due to lower subcutaneous and abdominal fat levels. The intramuscular fat, amino acid, and unsaturated fatty acid contents are vital indicators for evaluating meat quality [11]. Furthermore, the amount and type of taste-active fatty acids and amino acids play a decisive role in contributing to meat flavor [12]. To evaluate the effects of different rearing systems on the meat quality of Lueyang black chickens and to clarify the physiological and biochemical mechanism of the effects, transcriptome sequencing analysis of the breast muscles was used to determine the genes involved in meat quality. Following transcriptome analysis of the breast muscles, the DEGs (*GOS2*, *ASNS*, *NMRK2*, *GADL1*, *SMTNL2*, *SLC7A5*, *AMPD1*, *GLUL*) associated with meat quality were screened to further investigate the regulatory pathways related to meat quality formation. This experiment provided a novel and valuable theoretical basis for the rearing system selection and the functional molecular mechanisms associated with the meat quality of Lueyang black-bone chickens and other indigenous poultry.

## 2. Materials and Methods

### 2.1. Ethics Approval Statements

All poultry used in our study were approved by the Institutional Animal Care and Use Committee of Northwest Agriculture and Forestry University (Protocol number: #0806/2021 and 6 August 2021 of approval).

### 2.2. Animals, Diets and Experimental Design

The experiment was divided into three groups (cage, flat-net, and free-range), each group had three replicates, resulting in 100 individuals per replicate (half males and half females), and they were raised in Shaanxi Longjia Agricultural Co, Ltd. (Lueyang, Shaanxi, China). A total of 900, one-day-old healthy Lueyang black-bone chickens were used in the assessment. The one-day-old chickens were placed in cardboard boxes, then tagged with individually numbered leg rings and randomly distributed into three different rearing groups (caged, flat-net, and free-range groups). For the cage group, the birds were kept in cages (2 bird/cage, bird/0.15 m^2^). Each replication consisted of 50 adjacent cages. For the flat-net rearing group, the birds were kept in an area 70 cm above the ground on a stainless frame with a flat wire net covering. Each area was 800 cm × 625 cm and contained 100 birds (bird/0.5 m^2^). This group consisted of three identical areas. For the free-range group (100 birds/rearing area 100 m^2^, bird/1.0 m^2^, three identical rearing areas). From the starting period, the chicks were kept at 34–35 °C and then the temperature was gradually reduced by 3 °C per week until the final temperature of the rearing system reached 24 °C. When the temperature was higher than 24 °C, a ventilation system was used to reduce the temperature, and the temperature was raised by heating when it was lower than optimal, and all these efforts were in order to keep the animals in the optimal growth environment. The free-range rearing period was conducted during May and June, when the local average daily temperature was 22.5 °C. It was suitable for chicken growth. In order to ensure the health of the birds and improve their disease resistance, immunization vaccines were given on time at all stages in the growth of the Lueyang black-bone chickens.

All chickens were raised with the same amount and composition of diet under the three rearing systems and had free access to feed and water. The starter diet was used for the first six weeks, then replaced with a grower diet (Supplementary Table S1). Lueyang black-bone chickens are a precious breed protected by the local government. Due to the small number and the preciousness of Lueyang black-bone chickens, we randomly selected 12 birds (half males and half females) per group (a total of 36 chickens) for experimental research. All chickens were euthanized using carbon dioxide anesthesia and conventional neck cutting.

### 2.3. Measurement of Meat Quality Traits

In all carcasses, meat quality traits including pH, shear force, crude protein, fat, inosine monophosphate (IMP), and fatty acid content in breast muscles were measured. After slaughter, pH was measured 1 h post-mortem on the breast muscles using a pH meter (ModelPHS-3E; Leici, Shanghai, China). The samples were cut into strips of 2 cm (length) × 1 cm (thickness) × 1 cm (width), and then the muscle fibers were sheared perpendicular using a Zhiqu shear device (Model ZQ-990L; Zhiqu, Guangdong, China). Recording the maximum force (N) was required to pass the sample. The pH and shear force were measured five times in parallel. The intramuscular fat and crude protein contents were measured using a FOSS NIR Rapid Meat Analyzer (Food Scan2; Hillerod, Denmark). The amino acid and fatty acid compositions were determined according to the previous study [13,14] (Supplementary Material S1). The amount of IMP, an important umami component in chickens, was measured using HPLC according to standard protocols [15].

### 2.4. Total RNA Extraction, cDNA Library Preparation, and Sequencing

After slaughter, the breast muscles from six randomly selected hens were dissected in the same area, and the tissues were immediately placed in liquid nitrogen. To minimize the effects of transcriptome variation among individuals, the RNA from the six hens was randomly mixed in pairs into three RNA samples from each group. Total RNA was extracted from the breast muscles using TRIzol reagent (Accurate Biology, Beijing, China) as previously described [15]. The RNA quality and amount were evaluated using agarose gel electrophoresis and Thermo Scientific NanoDrop 2100 (Thermo Fisher, Waltham, MA, USA), respectively. The RNA samples were treated with DNase for fragment, and mRNA was purified using oligo-dT magnetic beads. The isolated mRNA fragments were split into approximately 375-bp by using chemical reagents at a suitable temperature. The fragmented mRNA was detected with random hexamer primers, and then reverse transcribed with reverse transcription to produce cDNA, then the complementary strand of cDNA was synthesized, and then the mRNA was removed. The 3′-termini of purified cDNA fragments were repaired, and A-tails were added, and then T-linked to the sequencing splice and labeled. PCR was used to enrich and amplify the fragments with adaptors to obtain the RNA-seq library of Lueyang black-bone chicken muscle. Library sequencing was detected on an Illumina Noveseq (ThermoFisher, Shanghai, China), and the raw data were saved in FASTQ format. Before analyzing the experimental data, the Fast QC (http://www.bioinformatics.babraham.ac.uk/projects/fastqc/ accessed on 8 August 2021); software was used to evaluate the quality of the raw data obtained from sequencing, and the adaptors and low-quality reads were removed to finally obtain the clean reads. All clean reads were mapped to the chicken genome assembly of Gallus gallus, namely GRCg6a, using bowtie2/tophat2, and then compiled and evaluated [16].

### 2.5. Bioinformatic Analyses of DEGs

The normalized expression abundance of each assembled transcript was detected using fragment FPKM. For quantifying the abundance of assembled transcripts, all reads were mapped to a non-redundant set of transcripts. The FPKM threshold for gene detection was set at >1 [17]. Gene expression levels were directly used to compare DEGs. DEGs were identified using DESeq software (http://www-huber.embl.de/users/anders/DESeq/ accessed on 19 August 2021). Screening was performed with |log2 FC| > 1 and *p* adj < 0.05 as the criteria to obtain differentially expressed genes. A false discovery rate adjusted *p*-value < 0.05 and |log2 Fold Change| > 1 were used as the criteria to obtain DEGs. To provide a comprehensive and detailed interpretation of the functions and regulatory pathways involved in DEGs, GO and KEGG pathway were used to analyze genes with significant differences [18].

The FPKM values of genes expressed in the three groups were homogenized using the Z-score, and the clustered heat map was constructed using the P heatmap R package. Genes with a high correlation between the expression levels of the samples were classified into one group. Clustering was performed based on the expression levels of the same gene in different samples, and the expression patterns of different genes in the same sample. The Euclidean method was used to calculate distance, and the complete linkage method was used for clustering. The STRING database (http://string-db.org/, accessed on 8 October 2021) was used to construct the protein–protein interactions (PPI) network with the candidate DEGs, which may play important biological roles [19].

### 2.6. Real-Time Quantitative PCR (RT-qPCR) Verification

Eight candidate DEGs which relate to fat deposition, skeletal muscle function, and flavor formation were selected for RT-qPCR to prove the exact RNA-seq data. RT-qPCR was performed to detect the expression of target genes *GOS2*, *ASNS*, *NMRK2*, *GADL1*, *SMTNL2*, *SLC7A5*, *AMPD1*, and *GLUL* at mRNA level using cDNA as the template and β-actin gene as the internal reference gene. This experiment was repeated three times using a SYBR green kit (Invitrogen, Thermo Fisher, Waltham, MA, USA) on an ABI Step OnePlus system. The detailed PCR reaction procedure is described in Supplementary Material S2. Primers (Table 1) were designed using Primer 5 software (PREMIER Biosoft Lab, San Francisco, CA, USA) based on relevant gene sequences retrieved from the NCBI (https://www.ncbi.nlm.nih.gov/, accessed on 13 October 2021). There were three biological replicates per sample, and the expression of candidate DEGs was calculated using the 2^−ΔΔCt^ method.

### 2.7. Statistical Analyses

The results were expressed as means with a standard error of the mean (SEM). Data were analyzed using the SPSS 26.0 statistical software (SPSS, Chicago, IL, USA), and one-way ANOVA was used to analyze the effects of different rearing systems on meat quality parameters and amino acid composition, which was then followed by LSD multiple comparison tests. The significance level for all statistical analyses was set at *p* < 0.05.

## 3. Results

### 3.1. Meat Quality Parameters

Meat quality parameters from 16-week-old chickens under three different rearing systems are shown in Table 2. It is clear that the meat quality characteristics were influenced by the rearing system. The results showed that the values of pH, shear force, IMP, palmitic acid, and linoleic acid were the highest in free-range rearing among the three rearing systems. Meanwhile, the pH, shear force, IMP, linoleic acid, and palmitic acid contents in free-range rearing were significantly higher than those in caged rearing (*p* < 0.05), the pH and IMP in flat-net rearing were significantly higher than those in caged rearing (*p* < 0.05), while the shear force, linoleic acid, and palmitic acid were not significantly different between caged and flat-net rearing (*p* > 0.05). Fat content in caged rearing was significantly higher than that of the flat-net and free-range groups (*p* < 0.05). Meanwhile, the fat content in the flat-net group was also significantly higher than that of the free-range group (*p* < 0.05). As for the content of oleic acid, it was significantly higher in the flat-net group compared to that of the free-range and caged groups (*p* < 0.05), while there was no significant difference between caged and flat-net rearing (*p* > 0.05). Saturated fatty acids, stearic acid, and myristic acid showed no significant difference between the three treatments (*p* > 0.05).

The effects of different rearing systems on the amino acid composition of breast muscles are shown in Table 3. The levels of essential amino acid Threonine (Thr), Valine (Val), Methionine (Met), Isoleucine (Ile), Leucine (Leu), Lysine (Lys), and total essential amino acids in caged and flat-net rearing were significantly higher compared with free-range rearing (*p* < 0.05), whereas there was no significant difference between caged and flat-net rearing (*p* > 0.05). With regard to dispensable amino acid, the content of Aspartic (Asp), Alanine (Ala), and Tyrosine (Tyr) was significantly lower in the free-range group than in the other two groups (*p* < 0.05), and there was no significant difference between caged and flat-net rearing (*p* > 0.05). Additionally, the amount of umami amino acids was determined, and the total umami amino acids accumulated more in breast muscles in the freedom-restricted caged systems. Interestingly, as a major contributor to umami, the Glu content in free-range rearing was the highest among the three experiment groups, and significantly higher than the caged group (*p* < 0.05). Additionally, there was no significant difference between free-range and flat-net rearing (*p* > 0.05).

### 3.2. Sequencing of mRNA in Chicken Breast Muscle

**Figure 1:**
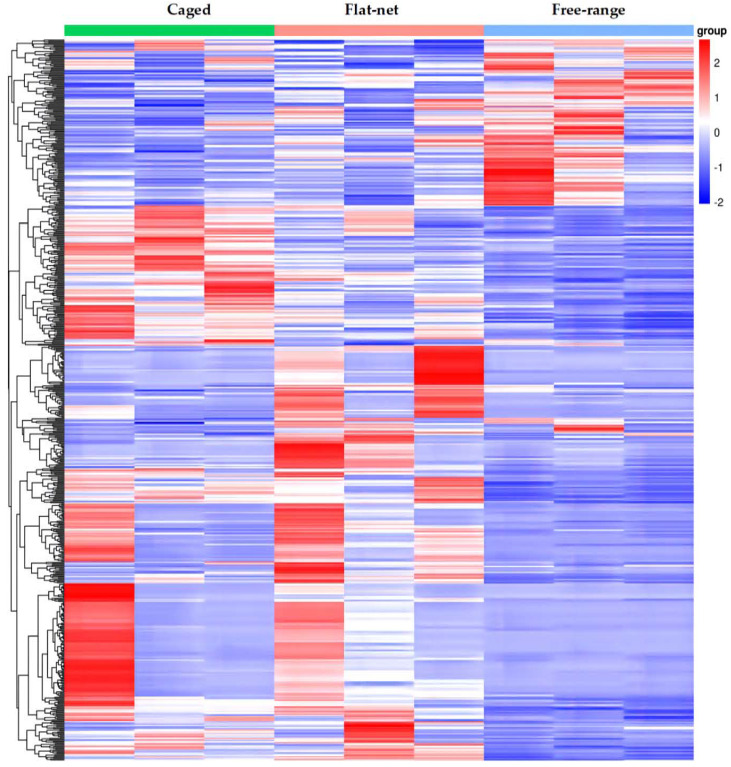
Gene expression clustering heat map under three rearing systems. Each column in the heat map represents the expression of different genes in the same sample, and each row represents the expression of the same gene in different samples. Red genes are up-regulated, and blue genes are down-regulated in the affected samples.

Sequencing raw reads were strictly filtered to remove sequences containing sequencing junctions and low-quality reads in order to obtain high-quality clean reads for bioinformatics analysis. A total of 3.59 × 10^8^ clean reads, with an average of 3.99 × 10^7^ reads per sample (ranging from 3.53 × 10^7^ to 4.24 × 10^7^), were obtained. The Q20 and Q30 quality values were 96.21% and 90.29%, respectively. After mapping to the Gallus genome, the average effective mapping ratio was 90.01%. Cluster analysis was used to determine the patterns of gene expression in the different rearing systems. As shown in Figure 1, the gene expression clustering of the free-range group displayed a reverse pattern compared to that of the caged and flat-net groups. After pairwise comparison of the three groups, 131, 387, and 291 DEGs were differently expressed in the three treatments (flat-net vs. caged, flat-net vs. free-range, and caged vs. free-range, respectively). Furthermore, the expression of *GOS2* was the highest, and that of *GADL1* was the lowest, in the free-range group (Table 4).

### 3.3. Enrichment and Interaction Analyses of DEGs

**Figure 2:**
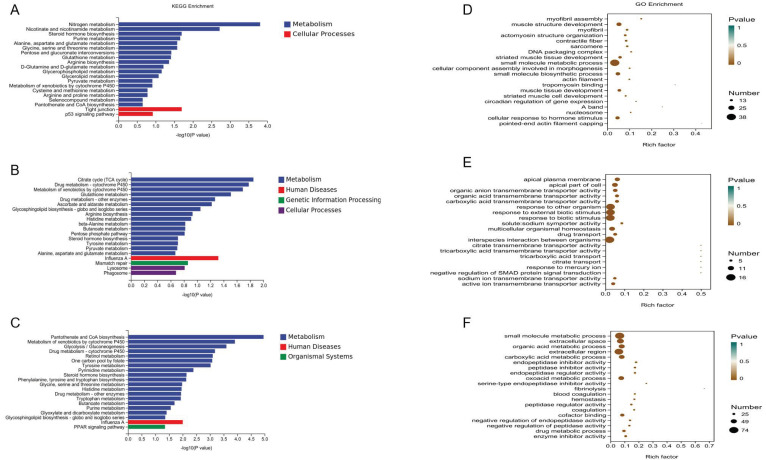
Top 20 GO and KEGG enrichment of DEGs. KEGG pathway (**A**–**C**) and GO term (**D**–**F**) enrichment were conducted with DEGs of three groups (caged vs. free-range, flat-net vs. caged, and flat-net vs. free-range). The vertical ordinate is the pathway name and the horizontal coordinate value is the ratio of enriched genes to total genes. The sizes of the point indicate how many DEGs are in the pathway, and the colors of different points correspond to different *p*-value ranges.

GO and KEGG enrichment analyses were conducted with DEGs of three groups (caged vs. free-range, flat-net vs. caged, and flat-net vs. free-range) (Figure 2). There was a total of 291 DEGs between the caged and free-range groups which were mainly enriched in amino acid metabolism pathways (metabolism of nitrogen, alanine, aspartate, glutamate, glycine, serine and threonine, and arginine biosynthesis) and muscle development terms (myofibril assembly, muscle structure, muscle tissue, striated muscle cell, and muscle cell development). Between the flat-net and caged groups, 131 DEGs were mainly enriched in stimulate response terms (response to other organisms, response to external biotic stimulus, response to biotic stimulus, and multicellular organismal homeostasis). A total of 387 DEGs between the flat-net and free-range groups were mainly enriched in amino acid metabolism (aromatic amino acid family catabolic process, tryptophan and histidine metabolism, and cellular amino acid metabolic processes), blood coagulation (regulation of blood coagulation and hemostasis, wound healing, and fibrin clot formation), and nucleotide metabolism (nucleoside phosphate, nucleoside monophosphate, nucleotide, and ribonucleoside metabolic processes).

**Figure 3:**
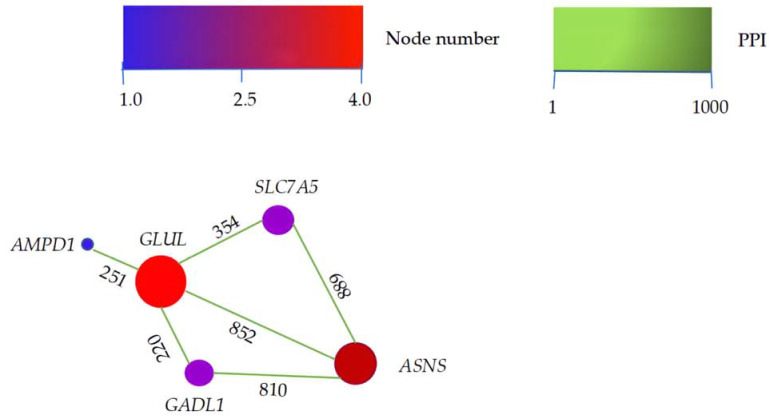
PPI network of candidate differently expressed genes. In the network, the higher the score of the interacting genes, the deeper the color of the lines between them, indicating a stronger interaction between two genes. The lines are directional, with the source gene pointing to the target gene along the direction of the number on the line. The larger the circle representing the gene, the more the interactions of the gene.

To investigate candidate DEGs that contacted with each other, the major DEGs were analyzed using STRING (v.11). Five flavor-related genes (*ASNS*, *GADL1*, *SLC7A5*, *AMPD1*, and *GLUL*) interacted with the candidate DEGs (Figure 3). *GLUL*, the hub of the PPI network, interacted with all other genes. As a target gene, *GLUL* had the strongest interaction (score = 852) with *ASNS* in the PPI network.

### 3.4. RT-qPCR Verification of Candidate Genes

**Figure 4:**
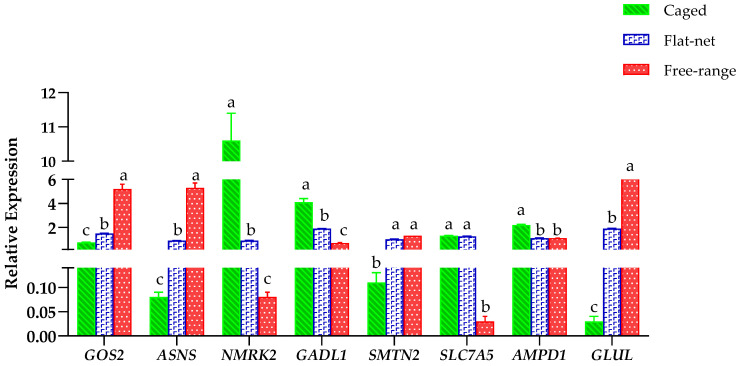
Validation of candidate DEGs under three rearing systems using real-time quantitative PCR. For each gene, different lowercase letters (a–c) indicate significant differences (*p* < 0.05).

Eight candidate DEGs (*GOS2*, *ASNS*, *NMRK2*, *GADL1*, *SMTNL2*, *SLC7A5*, *AMPD1*, and *GLUL*) that may be associated with different rearing systems were selected to validate the RNA-seq results using RT-qPCR (Figure 4). From the verification results, the eight candidate genes showed a transitional trend among the three rearing systems, which was consistent with our expectations. The expression of *GOS2*, *ASNS*, *SMTN2*, and *GLUL* was up-regulated for the caged, flat-net and free-range groups; meanwhile, the expression of *SLC7A5*, *NMRK2*, *GADL1*, and *AMPD1* was down-regulated. The expression of *GOS2*, *ASNS* and *GLUL* in the free-range group was significantly higher than the caged and flat-net groups (*p* < 0.05), while the flat-net group was significantly higher than the caged group (*p* < 0.05). The expression of *NMRK2* and *GADL1* in the caged group was significantly higher than the free-range and flat-net groups (*p* < 0.05), while the flat-net group was significantly higher than the free-range group (*p* < 0.05). Subsequently, the results of RT-qPCR validation using RNA-seq were compared. In summary, the expression of these candidate DEGs in Lueyang black-bone chickens may be affected by the three rearing systems.

## 4. Discussion

### 4.1. Meat Quality Parameters

Meat quality, which includes pH, shear force, amino acids, and fatty acids, is an important characteristic that determines the degree of acceptability for consumers. The pH is dependent on the amount of glycogen in the muscle. After slaughter, muscle pH is reduced when glycogen decomposes into lactic acid through the anaerobic pathway [20]. Meat products with a low pH result in poor meat quality and water-holding capacity [21]. In this study, the pH of the free-range group was significantly higher than that of the other two groups, which is consistent with previous studies, and it was concluded that the pH of meat products from outdoor, slow-growing broilers was higher than that of indoor, slow-growing broilers [22]. The reason for the high pH in free-range chickens may be related to the relatively low content of muscle glycogen, and the lower lactic acid through the anaerobic pathway when it decomposed [23]. Of all the sensory characteristics, tenderness is considered to be the most important by consumers, which means meat can be chewed or cut [24]. The slow-growing broilers assigned to the free-range group were much more active than those assigned to the other groups. This study found that the shear force of the muscles in the free-range group increased, and the meat was more compact from high-exercise conditions, which is consistent with the results published by Chen et al. [24]. The higher shear force in breast muscles of the free-range group is mainly attributed to the large range of motion and their frequent movements. However, Fu et al. [25] found that the rearing system did not affect the tenderness. This could be due to the difference in the extent of free-range rearing.

The flavor of meat is also determined by various flavor substances, such as IMP, unsaturated fatty acids, and flavor amino acids. IMP is a key factor in influencing fleshy flavor and is a major indicator of meat flavor [15]. For this reason, many scientists have focused their attention on the synthesis and degradation of IMP in different livestock and poultry [26]. In this study, black-bone chickens in the free-range and flat-net groups had the higher content of IMP. Zhang also showed that the free-range system contributed more to the deposition of IMP in muscles, by improving the expression levels of enzymes involved in IMP synthesis, thereby accelerating the de novo synthesis of IMP in chickens [15]. The effects of fatty acids on meat quality mainly depend on the content of unsaturated fatty acids [27,28]. More linoleic acid which belong to unsaturated fatty acids were contained in the free-range group, which could improve blood microcirculation by reducing blood viscosity, which is usually beneficial to human health. The higher unsaturated fatty acids in the free-range group may be associated with more diverse food sources in outdoor environments.

Moreover, the effect of amino acids on meat flavor is complex. The final flavor of meat is determined by the complex combination and proportion of multiple flavor amino acids rather than by a single type [29]. Interestingly, the total umami taste amino acid content was significantly higher in the caged group than in the free-range group. With less exercise, the levels of fat and crude protein increased in the caged group, which could bring health risks [25]. The flavor of the meat was significantly different between the three rearing systems, and transcriptome analysis was used to continue to investigate the specific mechanisms involved.

### 4.2. Regulatory Pathways Affecting Muscle Development and Flavor

Transcriptomic analyses are effective for studying animal production and have be-come an essential part of systems genomics or systems biology methods, and all the gene expressions in given tissues were provided with a snapshot of profiles, and insights into functional genes related to particular traits [30]. Different rearing systems can affect the quality of chicken meat, and different flavors can be observed. In order to investigate the factors affecting the flavor of chicken meat in depth at the genetic level, the transcriptional components and comparisons were performed in this study for chicken meat under three rearing systems. Using bioinformatics analysis, we screened genes and regulatory pathways related to influencing and regulating the flavor of meat. In the present study, there were 291, 131, and 387 DEGs among the three comparison groups (caged vs. free-range, flat-net vs. caged, and flat-net vs. free-range, respectively), which were mainly enriched in muscle development and amino acid metabolism pathways. These pathways may be related to the significant differences among the three different groups. In the comparison between the flat-net and caged groups, many stimulus-response-related pathways were enriched, which may be correlated to the fact that chickens experience more environmental stress in caged systems. DEGs in the flat-net vs. free-range groups were enriched in nucleotide metabolism pathways. The higher IMP content in the free-range group is also consistent with this result. Furthermore, in the comparison between the caged and free-range groups, the two most significant pathways were related to the formation of flavor substances and meat quality (small-molecule metabolic processes and muscle structure development). Small-molecule metabolic processes included nitrogen, alanine, aspartate, glycine, glutamate, threonine, serine metabolism, and arginine biosynthesis. Glutamate metabolism plays an important role in protein synthesis and is a mainly metabolic hub for many organisms. Nucleotide and cofactor biosynthesis, nitrogen assimilation, amino acid production, and secondary natural product formation are related to glutamate metabolism [30]. In particular, glutamate metabolism may play an important role in the differential content of Glu [31]. Skeletal muscle is an important tissue that maintains the normal functions of an organism, and it is also strongly correlated with important economic performance factors, such as weight [32]. Moreover, skeletal muscle is the principal tissue in the body, and without its function or regenerative properties, it will lead to debilitating musculoskeletal problems [33]. Therefore, these pathways may be involved in regulating the formation of flavored amino acids and muscle development in Lueyang black-bone chickens. Overall, these DEGs and pathways may play essential roles in regulating chicken flavor and quality.

### 4.3. DEGs on Muscle Development and Flavor

Eight candidate genes (*GOS2*, *ASNS*, *NMRK2*, *GADL1*, *SMTNL2*, *SLC7A5*, *AMPD1*, and *GLUL*), differentially expressed in the three comparison groups, were used to explore the molecular mechanism by which rearing systems influenced meat quality and flavor. Compared with the flat-net and free-range groups, the expression of *SLC7A5*, *NMRK2*, *GADL1*, and *AMPD1* was highest in the caged group. *SLC7A5*, a sodium-independent neutral amino acid transporter, affects cellular growth, metabolic homeostasis, and differentiation [34]. Additionally, the *SLC7A5* gene plays an important role in promoting tumor development [15]. *NMRK2*, a striated muscle-specific kinase, is required in muscle cells [35]. *NMRK2* deficiency leads to a greater need for muscles to reduce the energy required for contraction. Moreover, *NMRK2*-deficient mice more easily suffered muscle dysfunction in a knockout mouse study [36]. *GADL1* plays a role in the production of β-alanine and taurine, and is ultimately involved in the bio-synthesis of myostatin and pantothenate [37]. Previous studies on beef and geese have revealed that the high expression of *GADL1* improves growth performance, lipid metabolism, and meat quality [38,39]. *AMPD* is an important enzyme in the purine nucleotide degradation reaction, catalyzing the deamination of adenosine monophosphate to inosine monophosphate in skeletal muscle [15]. *AMPD1* expression was also positively correlated with IMP content and muscle flavor in chickens [40]. In the present study, these four genes were up-regulated in the caged group compared with the flat-net and free-range groups and may be important regulatory genes, which influenced higher levels of total amino acids and saturated fatty acids in the caged group.

*GOS2* plays a major role in fat deposition. *GOS2* is well-known for its function in lipolysis [41]. Even in the presence of CGI-58, the natural coactivator of adipose tri-glyceride lipase (ATGL), *GOS2* still inhibited hydrolase activity by interacting with the patatin structural domain of ATGL [42]. In the present study, *GOS2* was up-regulated in the free-range rearing group compared with that of the caged and flat-net rearing groups, and the higher expression of *GOS2* led to a lower fat content in the free-range group, which is beneficial to human health.

*ASNS* is ubiquitous in mammalian organs and its main biological function is glutamine dependent asparagine synthesis in an ATP-dependent reaction [43]. *ASNS* is associated with dysplasia and is down-regulated at the transcriptional level in the muscle tissue during compensatory growth [44,45]. The expression of *SMTNL2* is higher in skeletal muscle and may be contacted with differentiated myocytes [44]. *SMTNL* has been suggested to play essential roles in muscles by interacting with the function of the contraction regulators (e.g., myosin phosphatase, tropomyosin, and calmodulin) [46]. The expression of *ASNS* and *SMTNL2* in free-range rearing treatments was the highest among the three groups, which indicated that *ASNS* and *SMTNL2* play an important role in the production of amino acids and skeletal muscle development.

Glutamine synthetase, encoded by *GLUL*, converts glutamate and ammonia to glutamine. Previous studies have shown that *GLUL* can affect flavor development through taurine, pantothenate, IMP, and Glu [47]. In the present study, *GLUL* expression was significantly increased during free-range rearing, indicating that it plays an important role in umami amino acid production and muscle nutrition. In addition, as a hub gene in the PPI network, its expression in the free-range rearing group was significantly higher than that in the caged and flat-net groups, which proved that the free-range group may show a better flavor.

In summary, our findings revealed that different rearing systems affect the quality of meat by influencing the formation of flavor substances and gene expression, and revealed eight DEGs (*GOS2*, *ASNS*, *NMRK2*, *GADL1*, *SMTNL2*, *SLC7A5*, *AMPD1*, and *GLUL*) related to fat deposition, skeletal muscle function, and flavor formation. Due to the quantity of the samples, the experiment has some limitations. However, it still provides some suggestions for future investigations into the influence of different rearing systems on meat quality.

## 5. Conclusions

This is an original study on the meat quality, flavor, and regulatory pathway of the breast muscles of Lueyang black-bone chickens under three different rearing systems. In conclusion, our results support that free-range and flat-net rearing could improve several important meat quality traits. The wider range of movements, which increased the activity of the birds, contributed to the development of muscle mass, reduced fat, and improved the deposition of flavor substances. Furthermore, this study screened for DEGs and explored the molecular mechanism of the meat formation through transcriptome analysis, GO, and KEGG pathways.

Collectively, the results of this study will help to explore the functions of meat quality-related genes, better understand the genetic mechanisms related to flavor genes, and provide a valuable theoretical basis for future rearing systems selection in the poultry industry.

## Figures and Tables

**Table 1:** Primer pairs used for real-time PCR of Lueyang black-bone chickens in this study.

Gene	Accession Number	Primers	Sequence (5′-3′)	Product Length (bp)
*β-actin*	NM_L08165.1	F	TGTGATGGACTCTGGTGATGGT	164
		R	TCTCGGCTGTGGTGGTGAAG	
*COS2*	XM_015299248.3	F	CGTTCTTCGGCGTGGTCAT	155
		R	CGACTTCTTGCTCTGCTCCA	
*ASNS*	XM_015281395.3	F	AAGGTGCTGACTGATGATGGA	270
		R	CGGATGTTGCTCTTCACTGTT	
*NMRK2*	XM_015299836.3	F	CTCTTCGACCTCCGCTACTT	154
		R	CGCTGTCCTCCATCTCCTT	
*GADL1*	XM_025148022.2	F	TGGCTAGATACCTTGTGGAAGA	244
		R	ACCTGGCGGAAGAAGTTGA	
*SMTNL2*	XM_040650160.1	F	CTCTGTCACTAAGCCTGTCTCT	157
		R	CACCAGTTCTCGCCTCCTT	
*SLC7A5*	NM_001030579.2	F	TGCTCTACGCCTTCTCCAAT	104
		R	ACGCAGCCACATCATACCA	
*AMPD1*	XM_004935004.4	F	AGCCTCGCCTCTCAATCTATG	280
		R	GATTCATCATCCACGCTGTCAA	
*GLUL*	NM_205493.1	F	CGTGCCTGTATGCTGGTGTGAA	274
		R	GCCTCCTCGATGTGCTTGAGAC	

**Table 2:** Meat quality parameters measured under three rearing systems of Lueyang black-bone chickens.

Parameters	Caged	Flat-Net	Free-Range	SEM	*p*-Value		
					Caged vs. Flat-Net	Caged vs. Free-Rang	Flat-Net vs. Free-Range
pH	5.31 ^c^	5.52 ^b^	5.67 ^a^	0.17	0.007	0.000	0.016
Shear force (kg)	18.98 ^b^	21.97 ^b^	30.68 ^a^	5.75	0.146	0.000	0.001
Crude protein (g/100 g)	26.52 ^a^	25.68 ^b^	26.00 ^ab^	0.16	0.036	0.173	0.394
Fat (g/100 g)	0.58 ^a^	0.43 ^b^	0.33 ^c^	0.06	0.036	0.004	0.041
IMP (mg/g)	1.92 ^b^	2.29 ^a^	2.40 ^a^	0.06	0.001	0.000	0.228
Palmitic acid (%) ^#^	25.88 ^b^	25.27 ^b^	27.87 ^a^	0.39	0.418	0.017	0.003
Stearic acid (%) ^#^	11.70	12.57	11.96	0.30	0.413	0.811	0.539
Myristic acid (%) ^#^	1.12	1.53	1.23	0.17	0.346	0.804	0.482
Oleic acid (%) *	40.30 ^bc^	42.57 ^a^	38.93 ^c^	0.51	0.031	0.171	0.002
Linoleic acid (%) *	19.13 ^b^	18.39 ^b^	21.72 ^a^	0.53	0.536	0.038	0.009
Palmitoleic acid (%) *	2.97 ^ab^	3.60 ^a^	2.58 ^b^	0.18	0.130	0.342	0.021

Values are presented as mean and SEM. Within the same line for each factor, different lowercase letters (a–c) indicate significant differences (*p* < 0.05). Parameters marked by ^#^ belong to saturated fatty acids, and those marked by * belong to unsaturated fatty acids.

**Table 3:** AA measured under three rearing systems of Lueyang black-bone chickens.

Itemsmg/g	Caged	Flat-Net	Free-Rang	SEM	*p*-Value		
					Caged vs. Flat-Net	Caged vs. Free-Rang	Flat-Net vs. Free-Range
Essential amino acid							
Threonine (Thr)	1.08 ^a^	1.09 ^a^	1.04 ^b^	0.007	0.399	0.005	0.001
Valine (Val)	1.21 ^a^	1.20 ^a^	1.09 ^b^	0.067	0.631	0.000	0.000
Methionine (Met)	0.72 ^a^	0.73 ^a^	0.68 ^b^	0.007	0.745	0.000	0.000
Isoleucine (Ile)	1.16 ^a^	1.15 ^a^	1.05 ^b^	0.015	0.726	0.000	0.001
Leucine (Leu)	1.99 ^a^	1.99 ^a^	1.85 ^b^	0.079	0.902	0.000	0.000
Phenylalanine (Phe)	0.96	0.94	0.94	0.006	0.265	0.092	0.530
Lysine (Lys)	2.18 ^a^	2.18 ^a^	2.05 ^b^	0.019	0.957	0.001	0.001
Total essential amino acid	9.29 ^a^	9.27 ^a^	8.70 ^b^	0.083	0.875	0.000	0.000
Dispensable amino acid							
Aspartic (Asp) ^#^	2.35 ^a^	2.36 ^a^	2.25 ^b^	0.016	0.656	0.001	0.001
Serine (Ser)	0.95 ^ab^	0.96 ^a^	0.93 ^b^	0.007	0.470	0.067	0.016
Glutamate (Glu) ^#^	3.65 ^b^	3.73 ^ab^	3.75 ^a^	0.010	0.152	0.046	0.717
Glycine (Gly)	1.11	1.10	1.07	0.013	0.874	0.235	0.299
Alanine (Ala)	1.46 ^a^	1.46 ^a^	1.35 ^b^	0.013	0.941	0.000	0.000
Tyrosine (Tyr)	0.87 ^a^	0.88 ^a^	0.85 ^b^	0.006	0.793	0.049	0.012
Histidine (His)	0.81	0.81	0.82	0.011	0.781	0.824	0.618
Arginine (Arg)	1.62	1.61	1.58	0.010	0.667	0.113	0.233
Proline (Pro)	0.76 ^ab^	078 ^a^	0.73 ^b^	0.009	0.395	0.222	0.048
Cystine (Cys)	0.22	0.22	0.22	0.003	0.396	0.830	0.292
Total dispensable amino acid	13.89	13.70	13.43	0.102	0.466	0.073	0.256
Total umami amino acid	15.35 ^a^	15.17 ^ab^	14.66 ^b^	0.119	0.489	0.016	0.063
Total amino acid	23.20 ^a^	23.18 ^a^	22.13 ^b^	0.162	0.954	0.002	0.002

Amino acids marked ^#^, belong to the umami amino acids. Values are presented as mean and SEM. Within the same line for each amino acid, different lowercase letters (a–c) indicate significant differences (*p* < 0.05).

**Table 4:** Differential expression statistics of candidate genes.

Gene	Flat-Net vs. Caged			Flat-Net vs. Free-Range			Caged vs. Free-Range		
	Differentiate	Log2 FC	*p*	Differentiate	Log2 FC	*p*	Differentiate	Log2 FC	*p*
*GOS2*	Up	1.01	*	Down	−1.38	*	Down	−2.38	*
*GADL1*	Down	−1.24	*	Up	2.32	*	Up	3.56	*

* indicates a significant difference (*p* < 0.05).

## Data Availability

All data which support this study are available from the corresponding author.

## Supplementary Materials

The following supporting information can be downloaded at: https://www.mdpi.com/article/10.3390/genes13101898/s1. Table S1. Ingredients and chemical composition of diets for Lueyang black-bone chickens; Material S1. The amino acid and fatty acid compositions measured; Material S2. The detailed PCR reaction procedure.
