# Supplementary material for: Effects of Different Rearing Systems on Lueyang Black-Bone Chickens: Meat Quality, Amino Acid Composition, and Breast Muscle Transcriptome
Source: Genes (Basel). 2022 Oct 19;13(10):1898. doi: 10.3390/genes13101898 (PMC9601429; doi:10.3390/genes13101898)
Supplement: Supplementary file 1 [file genes-13-01898-s001.zip › Supplementary materials.pdf]

Table S1. Ingredients and chemical composition of diets for Lueyang black-bone chicken.

Items	Starter diet	Grower diet
Ingredients, %		
Corn	63.80	71.23
Wheat bran	8.62	2.47
Soybean meal	15.00	18.00
Corn protein powder	9.82	4.58
$CaHPO_4$ (anhydrous)	1.10	1.58
Limestone	0.30	0.59
NaCl	0.26	0.37
Premix[1]	1.00	1.00
L-lysine	0.05	0.10
DL-methionine	0.05	0.08
Total	100	100
Nutrient analysis, %		
CP	19.0	28.5
Crude fiber	8.0	10.0
Crude ash	8.0	25.0
Crude fat	4.6	4.2
Total calcium	0.8-1.5	2.5-5.5
Total phosphorus	0.50	0.90
Dry matter	86.0	88.0
Fatty acids, %		
Lauric acid (C12:0)	0.30	0.36
Myristic acid (C14:0)	0.26	0.48
Palmitic acid (C16:0)	22.34	21.83
Stearic acid (C18:0)	2.52	2.50
Oleic acid (C18:1n-9)	28.68	36.24
Linoleic acid (C18:2n-6)	39.20	31.43
a-linolenic acid (C18:3n-3)	2.44	2.30
Others	4.26	4.86
Total	100	100
digestible AA[2], %		
Met	1.51	1.62
Lys	2.21	2.25

[1]The premix supplied the following per kg of feed: vitamin A, 10,000 IU; vitamin D, 32,500 IU; vitamin E, 30 mg; vitamin K1, 2.5 mg; vitamin B1, 2 mg; vitamin B6, 3.8 mg; vitamin B12, 10 μg; D-pantothenic acid, 12 mg; folic acid, 0.80 mg; biotin, 110 μg; nicotinic acid, 40 mg; choline, 460 mg; manganese, 60 mg; iron, 40 mg; copper, 10 mg; zinc, 55 mg; iodine, 1.6 mg; and selenium, 0.35 mg.

[2]Digestible amino acid coefficients for ingredients were determined by Near-Infra Red spectroscopy (Thermo Fisher Antaris II, Waltham, USA) standardized with Evonik AMI-NONIR Advabced calibration.

Material S1 The amino acid and fatty acid compositions measured.

Amino acid content of the meats was determined following the published procedure, using an L-8900 amino acid analyzer (HITACHI, Tokyo, Japan). Briefly, approximately 100 mg of breast muscle sample was ground into a slurry with a high-speed universal crusher. Then, the meat slurry was transferred a glass bottle, and 10 mL of 6 mol HCl was added. After filling with nitrogen, the mixture was hydrolyzed at 110C for 22h. Subsequently, the hydrolysate was transferred into a 50 mL volumetric flask and diluted to calibration tail with ultrapure water.

Fatty acid composition of the breast muscle samples was determined following the previous method, using a Thermo Fisher Trace 1310 ISQ (Thermo fisher, Waltham, USA). Briefly, the extracted lipids were placed in a glass, and an appropriate amount of KOH-methanol ($C = 0.5 \text{ mol } L^{-1}$) was added for hydrolysis. After shaking for 1 min, the mixture was reacted in 95℃ water for 10 min, and the glass bottle was gently shaking all the time to obtain a free fatty acid mixture. The 7 mL BF3–methanol solution (W = 15%) was added to esterified the free fatty acid mixture through continuous agitation. After shaking for 10 s, put the glass in 80°C water for 20 min. Then adding 20 mL n-hexane, shaken the mixture for 2 min and then centrifuged at 3000 rpm for 15 min. Furthermore, fatty acids were separated and identified using a GC-2010+ gas chromatograph (Shimadzu, Kyoto, Japan).

Material S2 The detailed PCR reaction procedure.

The detailed PCR reaction procedure is described as follows. The 12.5 μL PCR reaction included 6.25 μL SYBR Premix Ex Taq II (TaKaRa, Dalian, China), 0.25 μL (10 pmoL/μL) specific forward primer, 0.25 μL (10 mol/μl) reverse primer, 0.5 μL ROX reference dye, 0.25 μL (10 ng/μL) diluted cDNA, and 5.25 μL RNase-free water. The cycling parameters were 95 °C for 10 min, followed by 37 cycles of 95 °C for 15 s, 57 °C for 30 s, and 72 °C for 45 s.
